# Self-medication with Antimicrobial Drugs in Europe

**DOI:** 10.3201/eid1203.050992

**Published:** 2006-03

**Authors:** Larissa Grigoryan, Flora M. Haaijer-Ruskamp, Johannes G.M. Burgerhof, Reli Mechtler, Reginald Deschepper, Arjana Tambic-Andrasevic, Retnosari Andrajati, Dominique L. Monnet, Robert Cunney, Antonella Di Matteo, Hana Edelstein, Rolanda Valinteliene, Alaa Alkerwi, Elizabeth A. Scicluna, Pawel Grzesiowski, Ana-Claudia Bara, Thomas Tesar, Milan Cizman, Jose Campos, Cecilia Stålsby Lundborg, Joan Birkin

**Affiliations:** * University Medical Center Groningen, Groningen, the Netherlands;; † University of Linz, Linz, Austria;; ‡ Vrije Universiteit Brussel, Brussels, Belgium;; § University Hospital for Infectious Diseases, Zagreb, Croatia;; ¶ Charles University, Prague, Czech Republic;; # Statens Serum Institut, Copenhagen, Denmark;; ** Health Protection Surveillance Centre, Dublin, Ireland;; †† Consorzio Mario Negri Sud, Chieti, Italy;; ‡‡ Ha'Emek Medical Center, Afula, Israel;; §§ Institute of Hygiene, Vilnius, Lithuania;; ¶¶ Directorate of Health, Luxembourg, Luxembourg;; ## St Luke's Hospital, G'Mangia, Malta;; *** National Institute of Public Health, Warsaw, Poland;; ††† Max Planck Institute for Demographic Research, Rostock, Germany;; ‡‡‡ Comenius University, Bratislava, Slovakia;; §§§ University Medical Centre, Ljubljana, Slovenia;; ¶¶¶ Instituto de Salud Carlos III, Madrid, Spain;; ### Karolinska Institutet, Göteborg, Sweden;; **** Nottingham City Hospital, Nottingham, United Kingdom

**Keywords:** Self-medication, antimicrobial agents, cross-sectional studies, Europe, drug resistance, bacterial, drug utilization, research

## Abstract

We surveyed the populations of 19 European countries to compare the prevalence of antimicrobial drug self-medication in the previous 12 months and intended self-medication and storage and to identify the associated demographic characteristics. By using a multistage sampling design, 1,000–3,000 adults in each country were randomly selected. The prevalence of actual self-medication varied from 1 to 210 per 1,000 and intended self-medication from 73 to 449 per 1,000; both rates were high in eastern and southern Europe and low in northern and western Europe. The most common reasons for self-medication were throat symptoms (e.g., dry, inflamed, red, or sore throat, inflamed tonsils, tonsil pain). The main medication sources were pharmacies and medication leftover from previous prescriptions. Younger age, higher education, and presence of a chronic disease were associated with higher rates of self-medication. Attempts to reduce inappropriate self-medication should target prescribers, pharmacists, and the general public.

## Abstract 2

Antimicrobial drug self-medication occurs most often in eastern and southern Europe and least often in northern and western Europe.

Antimicrobial drug resistance is a rapidly increasing global problem (*1**,**2*), and prevalence varying widely among countries (*3*). Prevalence of resistance is positively correlated with prescribed outpatient drug use on a national level (*4**,**5*). However, actual consumption of drugs may also include self-medication, i.e., using drugs obtained without prescription. Other sources of self-medication may include leftover drugs from treatment courses prescribed earlier or drugs obtained from relatives or friends. Use without medical guidance is inappropriate because using insufficient dosages or incorrect or unnecessary drugs increases the risk of the selection of resistant bacteria (*6*) and the spread of antimicrobial drug resistance (*7*).

To date, the information on self-medication with antimicrobial drugs in the industrialized world is limited. In the United States, several studies indicate considerable use of leftovers (*8**–**10*), drugs obtained from a family member, a pharmacy, or a source outside the country (*11**,**12*). For example, in an Hispanic neighborhood of New York City, antimicrobial drugs are available without a prescription (*13*). In Europe, studies describing self-medication and storage of antimicrobial drugs in Spain (*14**,**15*), Greece (*16**,**17*), Russia (*18*), and Malta (*19*) also suggest considerable use of the drugs without consulting a physician. However, these studies were small or used selected samples and were not carried out in northern and western Europe. Moreover, because of the different research methods used, no meaningful comparison between countries was possible. In addition, little information exists on factors that puts person at-risk for self-medication. This survey was designed to fill that gap for 19 European countries: Austria, the Netherlands, Sweden, United Kingdom, Ireland, Denmark, Italy, Malta, Luxembourg, Belgium, Spain, Israel, Romania, Czech Republic, Slovakia, Lithuania, Slovenia, Croatia, and Poland. The aim of this study was to estimate and compare the prevalence of actual self-medication and at-risk for self-medication with antimicrobial drugs in participating countries. The demographic characteristics associated with such use, the types of drugs used, the sources of self-medication, the symptoms for which the drugs were reportedly used, and duration of use were also examined.

## Methods

Countries participating in the study were recruited from 2 networks of surveillance systems: European Surveillance of Antimicrobial Consumption (*20*) and European Antimicrobial Resistance Surveillance System (*21*). A multistage sampling design was used for sample selection in each participating country. Within each country, a region with average prescribed antimicrobial drug consumption was chosen. In those countries where this information was not available (Poland, Czech Republic, Lithuania, Croatia, and Romania), a region was selected that was representative of the country's population in terms of age and sex. In each region, a city (75,000–750,000 inhabitants) and a rural area (5,000–10,000) were selected. By using population registries, including lists of persons in the identified cities and rural areas, persons >18 years of age were selected by simple random sampling (computer-generated random numbers). To calculate the sample size, we needed the standard deviation of the unknown prevalence. As the standard deviation was a function of this unknown prevalence, we took the maximal possible value of the standard deviation (*22*). To obtain a precision of 0.05, the sample size needed was 400 persons per country. To adjust for possible nonresponse, we selected larger samples; sample sizes in the countries were 1,000–3,000 persons, equally distributed in urban and rural areas. Self-administered questionnaires were mailed between March and July 2003, and reminders with a new questionnaire attached were sent 2–4 weeks later.

We developed an English questionnaire specifically for this survey, translated it into national languages, and back-translated it to ensure consistency.^1^ The questionnaire was pilot-tested in each country. It could be completed either anonymously or with identifiable details to allow a follow-up study. Questions asked about the respondent's use of antimicrobial drugs during the past 12 months, how they were obtained, how they were stored at home, and whether the respondent would consider using drugs without consulting a physician. Details of the drugs used (name of the medicine, symptom or disease coded with International Classification of Primary Care codes [23], and duration of use) and demographic characteristics of the respondents were included. Antibacterial drugs for systemic use (Anatomical Therapeutic Chemical class J01) (*24*) were included in the analyses. Medicines erroneously reported as antimicrobial drugs were excluded from the analyses. Ethics or data committee approval for the survey was required in Sweden, Denmark, Belgium, United Kingdom, Ireland, Malta, Czech Republic, Slovenia, Croatia, Romania, and Lithuania and was obtained from the local ethics or data committees of these countries.

Respondents were classified as self-medicating if they reported that they had taken any antimicrobial drugs in the previous 12 months without a prescription from a physician, dentist, or nurse and as prescribed users if antimicrobial drugs had been prescribed. (Physician respondents who reported using nonprescribed drugs were not classified as self-medicating.) Those classified as at-risk for self-medication included those who indicated the intention to self-medicate or store drugs at home. Intended self-medication was defined as answering "yes" or "maybe" to the question, "In general, would you use antimicrobial drugs for yourself without contacting a doctor/nurse/hospital?" Two estimates were used to assess storage of drugs: a maximum estimate, including all respondents who stored antimicrobial drugs, and a conservative estimate that excluded those respondents who stored antimicrobial drugs and had taken the same drugs for a prescribed course in the previous 12 months.

### Statistical Analyses

Descriptive statistics were used to estimate the prevalence rates per 1,000 respondents and 95% confidence intervals (CI) for actual self-medication and prescribed use in the previous 12 months and for at-risk self-medication in each country. To assess possible bias from low response rates, we also estimated adjusted prevalence rates. We applied the continuum of the resistance model (*25*), based on the assumption that late respondents most resemble nonrespondents. Late respondents in our study were those who replied after the reminder. The adjusted prevalence is considered similar to the observed prevalence when it falls in the 95% CI of the observed prevalence.

The effects of individual characteristics and country on antimicrobial drug self-medication were studied with logistic regression analyses by using 3 outcome variables: actual self-medication in the previous 12 months, storage of antimicrobial drugs, and intended self-medication. Countries were grouped together in 3 European regions: northern and western (Sweden, Denmark, the Netherlands, Austria, Belgium, Luxemburg, United Kingdom, and Ireland), southern (Malta, Italy, Israel, and Spain), and eastern (Czech Republic, Slovenia, Croatia, Poland, Slovakia, Romania, and Lithuania). This grouping was based on patterns of prescribed use of antimicrobial drugs (*4*), geographic location (*26*), similarities in healthcare systems, and socioeconomic development. The former socialist countries are referred to as eastern countries. We tested possible interactions between the factors found to be significant and set the significance at p<0.01 for interaction terms due to multiple testing. Multivariate logistic regression was also used to study the relationship between intended self-medication, storage, and actual self-medication in the previous 12 months. Data were analyzed by using SPSS (version 11) for Windows (SPSS Inc, Chicago, IL, USA).

## Results

A total of 15,548 respondents completed the questionnaires. The mean response rate of the countries was 40% (Table 1).

**Table 1:** General characteristics of respondents in each European country

Country	Response rate (%)	No. respondents	Mean age (y) ± SD	Female (%)	Low education level* (%)	Presence of chronic disease (%)†	Urban location (%)
Northern and western							
The Netherlands	55	1,634	48 ± 17	58	30	15	48
Sweden	69	704	54 ± 19	53	53	14	49
Denmark	63	1,881	48 ± 17	56	60	14	49
Austria	28	442	49 ± 16	50	64	15	53
Belgium	54	1,734	45 ± 16	55	32	13	50
Luxembourg	50	675	46 ± 18	51	49	15	45
United Kingdom‡	23	675	50 ± 10	58	40	15	58
Ireland	26	793	48 ± 16	59	53	17	47
Southern							
Israel	18	467	50 ± 17	61	19	22	36
Malta	54	541	46 ± 16	55	60	21	47
Italy	21	213	45 ± 18	61	37	27	51
Spain	20	204	47 ± 14	47	31	18	57
Eastern							
Czech Republic	59	1,169	54 ± 15	36	45	27	48
Slovenia	38	1,143	48 ± 17	58	70	20	47
Croatia	31	615	53 ± 16	55	10	26	58
Poland	32	935	45 ± 18	60	42	23	52
Slovakia	55	546	41 ± 16	54	27	23	54
Romania	43	430	50 ± 18	49	43	27	43
Lithuania	25	747	59 ± 18	35	32	39	54

*Low education level was defined as incomplete primary education, completed primary education, and lower vocational or general education. †Including any of the following diseases: asthma, chronic bronchitis, emphysema, human immunodeficiency virus infection, cystic fibrosis, diabetes, endocarditis, tuberculosis, prostatitis, chronic urinary tract infection, chronic osteomyelitis, peptic ulcer disease, chronic pyelonephritis or cancer. ‡Reminders were not sent to nonrespondents.

### Prevalence of Self-medication and Prescribed Use

The prevalence rates of antimicrobial drug self-medication (actual and at-risk) and prescribed use are presented separately for countries with response rates >40% and <40% (Table 2). In both of these groups, prevalence rates for actual self-medication were highest in eastern (in particular, Romania and Lithuania), followed by rates in southern (Malta, Spain, and Italy) Europe. The lowest rates were in northern and western (the Netherlands and Sweden) Europe. The rates of at-risk self-medication also tended to be higher in southern and eastern Europe than in northern and western Europe. The adjusted estimates of prevalence rates of self-medication were similar to the observed rates for many of the countries.^2^ In Luxembourg, Austria, Israel, Spain, and Lithuania, the adjusted rates of self-medication were consistently higher than the observed rates, indicating that the observed rates may underestimate the prevalence in these countries. By contrast, in Romania, Croatia, and Slovenia, the adjusted rates were lower than the observed rates, indicating that the prevalence rates might be overestimated in our study.

**Table 2:** Actual use of systemic antimicrobial drugs in the last 12 months and at-risk self-medication in 19 European countries

Country (region in country)	Rate per 1,000 respondents (95% confidence interval)				
	Actual self-medication	Prescribed use	Intended self-medication	Storage* (conservative estimate)	Storage† (maximum estimate)
Countries with response rate >40%					
Northern and western					
	The Netherlands (Twente)	1 (0.2–4)	152 (134–170)	85 (71–101)	10 (6–17)	36 (28–46)
	Sweden (Vastmanland)	4 (0.9–12)	135 (109–161)	118 (94–143)	14 (7–26)	43 (29–60)
	Denmark (Funen, Aarhus, Copenhagen‡)	7 (4–12)	172 (154–189)	132 (116–147)	42 (33–52)	84 (72–97)
	Luxemburg (whole country)	9 (3–19)	288 (252–324)	83 (62–107)	90 (69–114)	132 (106–158)
	Belgium (East Flanders, Flemish Brabant)	9 (5–15)	222 (201–242)	80 (67–95)	71 (59–84)	123 (107–138)
Southern					
	Malta (whole country)	56 (38–79)	422 (380–465)	228 (192–264)	156 (125–186)	269 (232–306)
Eastern					
	Czech Republic (Hradec Krlov)	7 (3–13)	253 (228–279)	179 (156–201)	45 (33–58)	64 (51–80)
	Slovakia (Middle Slovakia region)	42 (27–63)	569 (527–612)	324 (284–365)	192 (159–225)	302 (263–340)
	Romania (Dolj)	198 (160–235)	307 (263–351)	431 (383–478)	200 (162–238)	321 (277–365)
Countries with response rate <40%§					
Northern and western					52 (33–77)
	Austria (Upper Austria)	9 (2–23)	159 (124–195)	73 (49–103)	34 (19–55)	52 (33–77)
	United Kingdom (Nottinghamshire)	12 (5–23)	221 (189–254)	166 (137–195)	33 (21–49)	74 (56–97)
	Ireland (Cork)	14 (7–25)	353 (320–386)	150 (125–176)	29 (19–43)	100 (80–123)
Southern					
	Israel (Northern Israel)	15 (6–31)	330 (287–374)	187 (150–223)	120 (91–149)	236 (197–274)
	Italy (Abruzzo)	62 (33–103)	512 (444–580)	243 (185–301)	379 (314–445)	569 (502–636)
	Spain (autonomous community of Madrid)	152 (103–201)	315 (251–379)	314 (249–380)	260 (200–320)	500 (431–569)
Eastern					
	Slovenia (Ljubljana region)	17 (10–26)	293 (266–320)	280 (253–307)	119 (100–137)	183 (160–205)
	Croatia (Zagreb county)	31 (19–48)	439 (399–478)	205 (172–237)	130 (103–156)	212 (179–244)
	Poland (Pomorskie)	33 (23–47)	199 (172–225)	115 (94–136)	69 (53–87)	137 (115–160)
	Lithuania (Klaipeda, Rietavas)	210 (181–239)	275 (243–308)	449 (412–486)	177 (149–204)	333 (299–367)

*Included only those respondents who stored antimicrobial drugs and had not taken the same antimicrobial drugs for a prescribed course in the previous 12 months. †Including all respondents who stored antimicrobial drugs. ‡Although Copenhagen has population >750,000, both self-medication and prescribed use of antimicrobial drugs were not significantly different between the sample of Copenhagen and sample of the other 2 Danish counties (χ^2^ tests). §The rates for these countries should be interpreted as first rough estimates.

We compared our estimates of antimicrobial drug self-medication with data available from the European Union's Eurobarometer survey in October 2002 (*27*). We calculated the prevalence of drug use from "leftovers" and drugs "directly from the pharmacy" by using the Eurobarometer data and compared these figures with the same estimates in our study. The estimates were similar with overlapping 95% CIs (data not shown) for countries with both high and low response rates. Our figures differed regarding Spain, for which we found a higher prevalence of self-medication than did the Eurobarometer. Three other studies (*4**,**15**,**28*) indicated an even higher prevalence of self-medication in Spain than in our estimate.

### Types of Antimicrobial Drugs Used for Self-medication and Duration of Use

**Figure A1:**
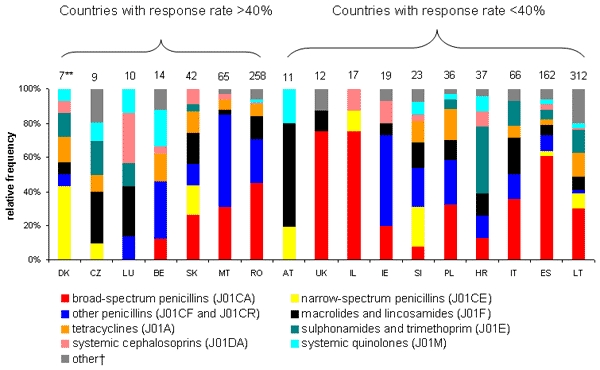
Use of major groups of antimicrobial drugs for actual self-medication in 17 countries; countries reporting less than 5 self-medication courses are excluded. DK, Denmark; CZ, Czech Republic; LU, Luxembourg ; BE, Belgium; SK, Slovakia; MT, Malta; RO, Romania; AT, Austria; UK, United Kingdom; IL, Israel; IE, Ireland; SI, Slovenia; PL, Poland; HR, Croatia; IT, Italy; ES, Spain; and LT, Lithuania. **Number of courses (taken in the previous 12 months) per 1,000 respondents per country. †Includes systemic amphenicols (J01B), aminoglycosides (J01G), combinations of systemic antibiotics (J01R) and other systemic antibiotics (J01X).

Antimicrobial drugs from all classes were used for self-medication in countries with response rates both >40% and <40% (information is shown in the Figure A1). Penicillins were the most commonly used, representing 54% of total courses in all countries. Among the countries with response rate >40%, southern and eastern countries used significantly more broad-spectrum penicillin for self-medication than northern and western countries (χ^2^, p<0.05). This difference was significant when the analysis was repeated and included all countries (χ^2^, p<0.01). Seventeen courses of self-medication with chloramphenicol in Lithuania and 1 course in the Czech Republic were found (data not shown). Ten courses of self-medication with parenteral (injectable) antimicrobial drugs, namely streptomycin or gentamicin, were found in Lithuania (data not shown). The median duration of actual self-medication was 5 days (1 to 100 days) and was significantly longer among the respondents who had chronic diseases (Mann-Whitney U test, p<0.01).

### Reasons for Self-medication and Sources

**Figure 1:**
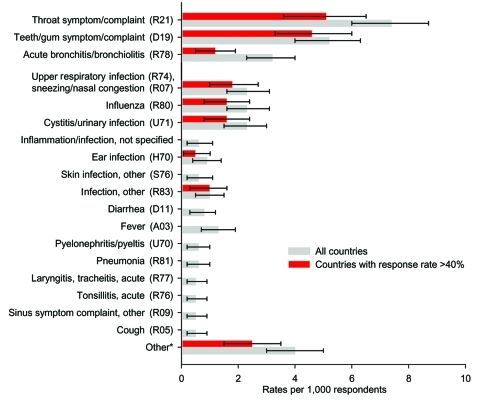
Prevalence of actual self-medication by symptoms or diseases classified by International Classification of Primary Care codes (rates pre 1,000 respondents and 95% confidence iinterval). *Symptoms or diseases with rates <1 per 1,000 respondents, including eye infection, pain, prostatitis, urogenital infection, headache, and "bad health."

A throat symptom (including red or sore throat), teeth or gum symptoms, and bronchitis were the most common reasons for self-medicating (Figure 1). Eye infection, pain, prostatitis, urogenital infection, headache, and "bad health" were among the other reasons for self-medication (data not shown). In countries with response rates >40%, a throat symptom was also the most common, followed by symptoms of the teeth or gums (Figure 1). Symptoms such as inflammation, skin infection, or diarrhea were reported only in countries with lower response rates. Self-medication for pyelonephritis or pyelitis was reported only in Lithuania; diarrhea was reported in Lithuania (10 patients; 9 used chloramphenicol), Austria (1 patient), and United Kingdom (1 patient).

**Figure 2:**
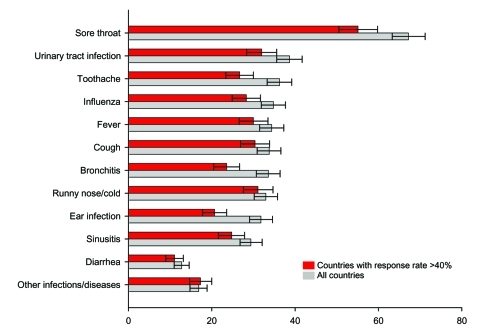
Prevalence of intended self-medication per predefined symptom (rates per 1,000 respondents and 95% confidence interval).

For intended self-medication as with actual self-medication, a sore throat was the most common symptom, followed by urinary tract infection and toothache (Figure 2). When including only those countries that had response rates >40%, sore throat and urinary tract infection were also the most common symptoms (Figure 2).

In eastern countries, the major source of self-medication was the pharmacy without prescription (309 courses, 68%), followed by leftover medications (120 courses, 26%). By contrast, in southern, northern, and western countries use of leftover medication was more prevalent (46 courses [51%] in southern countries and 35 courses [44%] in northern and western countries), followed by medications obtained directly from the pharmacy (41 courses [46%] in southern countries and 15 courses [19%] in northern and western countries). Among other sources of self-medication were drugs obtained from relatives or friends (52 courses, 8%, in all countries), drugs that were stored after being obtained abroad (10 courses, 2%, in all countries), and drugs obtained over the Internet (3 courses, all in Lithuania).

### Effects of Individual Characteristics

The effects of demographic characteristics and chronic disease on actual self-medication, intended self-medication, and storage of antimicrobial drugs are shown in Table 3. Sex and location (urban or rural) had no significant relevance in any of the 3 models. Respondents from southern and eastern countries were more likely to self-medicate (adjusted odds ratio [OR] 6.8, 95% CI 4.8–9.7, and 7.5, 5.7–10.0, respectively) than respondents from northern and western countries. Younger age, higher educational level, and presence of a chronic disease were all significantly associated with self-medication. Similar results were obtained for the relationship between demographic characteristics and storage of antimicrobial drugs, by using the conservative estimate of storage. Younger age, higher educational level, and presence of a chronic disease were also significant predictors of intended self-medication. Presence of a chronic disease increased the risk of intended self-medication, but this effect diminished with increasing age. We repeated all analyses including only those countries that had response rates >40% and obtained similar results. We also repeated these analyses separately for early and late respondents and obtained similar results.^3^

**Table 3:** Effects of individual characteristics on actual and at-risk antimicrobial drug self-medication*

Characteristics	Adjusted odds ratio (95% confidence interval)		
	Actual self-medication	Storage (conservative estimate)	Intended self-medication
Age	0.985 (0.979–0.992)	0.977 (0.973–0.982)	0.984 (0.980–0.987)
Region in Europe†			
	Northern and western	1 (reference)	1	1
	Southern	6.776 (4.752–9.662)	5.101 (4.240–6.137)	2.233 (1.909–2.613)
	Eastern	7.529 (5.676–9.985)	3.311 (2.868–3.822)	2.851 (2.577–3.154)
Education level			
	Low‡	1 (reference)	1	1
	High	1.357 (1.095–1.680)	1.690 (1.470–1.943)	1.233 (1.116–1.361)
Chronic disease§			
	No	1 (reference)	1	1
	Any	1.888 (1.497–2.383)	1.225 (1.038–1.446)	2.320(1.594–3.378)
	Age × any chronic disease			0.989 (0.982–0.996)
	Exponential (constant)	0.012	0.083	0.219

*At-risk self-medication included intended self-medication or storage of drugs at home. †Northern and western includes Sweden, Denmark, the Netherlands, Austria, Belgium, Luxemburg, United Kingdom, Ireland; southern includes Israel, Malta, Italy, and Spain; eastern includes Czech Republic, Slovenia, Croatia, Poland, Slovakia, Romania, and Lithuania. ‡Low education level was defined as incomplete primary education, completed primary education, and lower vocational or general education. §Including any of the following diseases: asthma, chronic bronchitis, emphysema, HIV infection, cystic fibrosis, diabetes, endocarditis, tuberculosis, prostatitis, chronic urinary tract infection, chronic osteomyelitis, peptic ulcer disease, chronic pyelonephritis, or cancer.

### Relation between Intended Self-medication, Storage, and Actual Self-medication

Intended self-medication and storage are both predictors of actual self-medication. A significant relationship was found between intended self-medication and storage. Intended self-medication was a strong predictor for actual self-medication for both respondents who stored drugs (OR 20.9, 95% CI 15.5–28.2) and those who did not (OR 17.8, 95% CI 14.0–22.7). However, for those who did not intend to self-medicate, storage also predicted higher actual self-medication (OR 3.5, 95% CI 2.2–5.6). When the analyses were repeated, including only those countries that had response rates >40%, similar results were obtained.^3^

## Conclusions

Self-medication with antimicrobial drugs occurred in all countries that participated in this survey. We included the data from both countries that had high and low response rates. In most of the countries with low response rates (except Spain), no other information is available about self-medication, an often overlooked issue. The second reason for including these countries was that low response was not a problem of this study only, but a general problem of surveys in these countries (*29**,**30*). This finding implies that if we want to include information about these countries, the results may be biased. In addition, debate is growing that low response is less problematic in affecting survey estimates than previously assumed (*31*). Nevertheless, the prevalence rates of self-medication in countries with low response rates should be considered as a rough estimate and interpreted as an indication that the problem exists.

Antimicrobial drug self-medication prevalence varies widely among different European regions, with the highest rates in eastern and southern countries, and the lowest in northern and western. Besides actual self-medication, intended self-medication is clearly relevant: it is a strong predictor of actual self-medication. Intended self-medication has a much higher prevalence than actual self-medication, indicating that the population at-risk is much larger than those who have actually self-medicated in the previous 12 months. Another risk factor for actual self-medication is the availability of drugs at home; opportunity encourages use. Our findings contribute to the growing evidence that estimates of antimicrobial drug use that are based on prescription data only are likely to underestimate actual consumption in both Europe and the United States (*11**,**32*). Our European estimates are low in comparison with those from a recent study in the US Hispanic community that showed that ≈20% of the respondents acknowledged getting drugs without a prescription in the United States (*32*). The only comparable high rates were found in Spain, Romania, and Lithuania, where they ranged from 9% to 18%. However, these figures should be compared with caution because our estimates refer to acquiring drugs without prescription in the last 12 months and the United States study refers to ever acquiring them.

We found that many persons used antimicrobial drug leftovers from previous prescriptions, as was the case in reports from the United States (*8**–**10**,**12*). Drugs could be left over because extra tablets were dispensed (in many countries pharmacies dispense drugs per package, not exact number of tablets) or because of patient noncompliance. Noncompliance may result in 2 inappropriate courses if the patient does not take the amount of medication prescribed and self-medicates later. Earlier findings indicated lower compliance in Italy and Spain than in Belgium, France, and the United Kingdom (*33*). In Italy, 41% of the interviewees who had taken drugs in the previous 12 months saved part of the course for future use, whereas only 4% of British interviewees reported this behavior (*33*).

In general, respondents' self-diagnosed disorders were self-limiting and antimicrobial drugs would not have been indicated. In contrast to studies in developing countries, this study identified few cases of self-medication for sexually transmitted diseases (*34**,**35*). Only 2 respondents in Lithuania reported self-medication for "gynecological infection" that might have been a sexually transmitted infection.

In this survey, persons who were more prone to self-medicate with antimicrobial drugs were younger persons, more educated, and had chronic diseases. This finding corresponds to those of studies conducted in the United States and Greece, which also found that higher educational status is associated with misuse of drugs (*8**,**17*). This relationship cannot be directly attributed to educational status. The interpretation of symptoms is also relevant. Previously, a study in the United States showed that persons with a higher education level tended to believe that antimicrobial drugs were less effective for upper respiratory infections with clear discharge but more effective with discolored discharge (*36*).

Antimicrobial drug self-medication is a cause for concern because it may contribute to the spread of antimicrobial drug resistance. Self-treatment with a drug that is ineffective against the causative organism or with an inappropriate dosage may increase the risk of selection of resistant organisms that are difficult to eradicate. These resistant organisms may then be transferred into the community. Our findings illustrate that adverse effects are aggravated by self-medication when unnecessary drugs, such as chloramphenicol, tetracycline, and aminoglycosides, are taken. Other problems related to self-medication include drug interactions, masked diagnoses, and superinfection.

Our results are comparable to those of other studies such as the Eurobarometer study (*14**,**19**,**27*). A study on antimicrobial drug storage among Spanish households showed that 42% of Spanish households had drugs at home, including those currently used (*14*). This finding is comparable to the prevalence of drugs stored (50%) in our study. In Malta, a higher prevalence (19%) of self-medication was found (*19*) than in our study, perhaps because the study included self-medication in the previous 2 years, while our study included the previous 12 months. Furthermore, the pattern of the prescribed use of drugs in different regions of Europe in our study is similar to that found in the study by Goossens et al., which was based on information from national databases (*4*).

A strength of our study is that we used the same methods and comparable samples in all countries, which facilitated an overview of the European situation. The low response rate in some countries is a limitation of our study, however. Although we calculated the prevalence rates adjusted for nonresponse, they are based on the assumption that respondents who replied after the reminder most resemble nonrespondents.

As with all self-reported data, results of this survey have the potential for recall bias, underreporting, or overreporting. We attached the list of the most commonly used antimicrobial drugs in each country to the questionnaires to reduce recall problems. To discourage underreporting of self-medication, the questions about drug use were formulated in a neutral way in which the source of the drug could be chosen from 6 predefined sources or "other source."

Substantial variation in the prevalence rates of antimicrobial drug self-medication among the European regions suggests that cultural (*37*) and socioeconomic factors play a role, as do disparities in health care systems such as reimbursement policies, access to health care, and drug dispensing policies. Another factor is the acquisition of antimicrobial drugs from pharmacies without prescription, which occurred most frequently in eastern European countries. Although over-the-counter sale of antimicrobial drugs is illegal in all participating countries, there is clearly a need to enforce the law in some countries.

Antimicrobial drug self-medication is a cause for concern in Europe. Even the lowest prevalence, 1 person per 1,000 respondents, implies that 10,000 persons in a population of 10,000,000 are self-medicating annually. Our study indicates a high prevalence of self-medication in countries that reported high resistance levels (southern and eastern countries). Even in the countries with low actual self-medication, substantial intended self-medication and drug storage occurs. Efforts to reduce inappropriate use of antimicrobial drugs should include the issue of self-medication and should involve prescribers, pharmacists, and the general public. The number of tablets dispensed in pharmacies should be limited, and patients should be instructed to discard their leftover drugs. Large-scale public campaigns, such as those recently launched in the United States, Canada, Belgium, and Australia (*38*), should include detailed instructions and emphasize the potential risks of using antimicrobial drugs without medical guidance.

## Footnotes

1 A copy of the questionnaire is available from the corresponding author on request.

2 The adjusted estimates for each country are available from the corresponding author on request.

3 Results are available from the corresponding author on request.
